# Gender and racial diversity among plenary session speakers at the Society of Abdominal Radiology Annual Meetings: a five-year assessment

**DOI:** 10.1007/s00261-022-03548-8

**Published:** 2022-05-21

**Authors:** Amani Shah, Elizabeth A. Sadowski, Kerry Thomas, Kathryn J. Fowler, Richard Kinh Gian Do, Sharon D’Souza, Parvati Ramchandani, Priyanka Jha

**Affiliations:** 1 Department of Radiology and Biomedical Imaging, University of California San Francisco, San Francisco, 505 Parnassus Avenue, Box 0628, San Francisco, CA 94143-0628 USA; 2 Department of Radiology, School of Medicine and Public Health, University of Wisconsin, Madison, WI USA; 3 Department of Radiology, University of North Carolina at Chapel Hill, Chapel Hill, NC USA; 4 Department of Radiology, University of California San Diego, San Diego, CA USA; 5 Department of Radiology, Memorial Sloan Kettering Cancer Center, New York, NY USA; 6 Tulsa Radiology Associates, Tulsa, OK USA; 7 Department of Radiology, Perelman School of Medicine, University of Pennsylvania, Philadelphia, PA USA

**Keywords:** Diversity, Plenary session speakers, Society of abdominal radiology

## Abstract

**Purpose:**

To evaluate the gender and racial diversity of plenary session speakers in the annual meetings of Society of Abdominal Radiology (SAR) over 2016 to 2020.

**Materials and methods:**

The brochures of the SAR annual meetings were reviewed for plenary session speakers and titles. Publicly available institutional profiles and social media were reviewed by the investigator in order to infer gender and race. Gender assessments were men, women, transgender men, transgender women or gender non-binary. Race was classified as White, Black or African American, American Indians and Alaskan Natives, Asian, Native Hawaiian and Pacific Islander and Multiracial. Statistical analysis was performed using chi square and *T*-tests.

**Results:**

Based on self-reported data, the SAR has 64% male and 36% female members. Over 2016–2020, plenary session speakers were more likely to be men [69.6% (183/263)] than women [30.4% (80/263)] (*p*-value = 0.0007). No speakers could be reliably identified as transgender, gender non-binary or gender expansive. In 2016, there were 24% women plenary speakers. This proportion was 28% in 2017, 33% in 2018 and 36% in 2019, and 30% in 2020. When assessing racial distribution, white speakers accounted for the majority of plenary speakers, ranging from 61 to 78%. Asians speakers accounted for 22 to 35%. There were no Black and African American, American Indian & Alaskan Native, Native Hawaiian & Pacific Islander plenary speakers (0%). Multiracial speakers were represented from 2018 to 2020, accounting for 2–4% speakers (*p*-value < 0.0001).

**Conclusions:**

Plenary speakers at SAR Annual Meetings from 2016–2020 were more likely to be men, but with the proportion of women presenters increasing over time. White speakers represented the majority of plenary session speakers, followed by Asians. No plenary session speakers were identified as Black or African American or Native Americans.

**Graphical Abstract:**

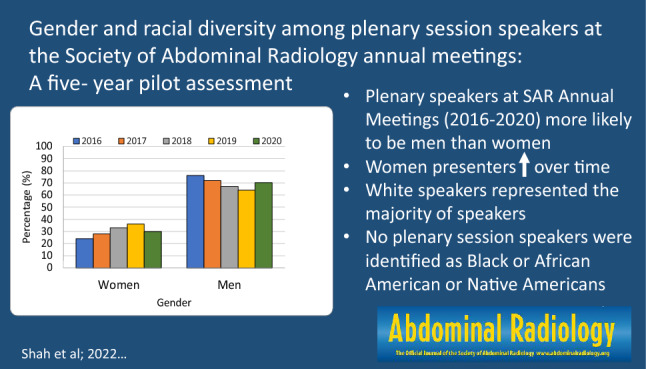

## Introduction

Workforce diversity is a crucial concern in medicine and radiology [1–3]. Underrepresented in medicine (URM) demographics account for only 8.3% of training and practicing radiologists, and less than 1/3 of radiologists are women [3]. Although the number of women entering medical schools nearly equals men, far less women choose radiology as their specialty [4]. Continued challenges related to recruitment, retention and promotion of women and URM faculty in radiology are likely multifactorial. These include lack of mentorship/sponsorship, microaggression, overt bias and low visibility of URM faculty in leadership positions may all contribute to the persistent underrepresentation of women and other underrepresented groups [1, 5, 6]. Plenary sessions at professional society meetings are a high visibility opportunity for the speakers [7, 8]. The sessions are important to both advance the careers of the speakers and for providing role models to women and URM participants in professional societies [7, 8].

Several recent publications have highlighted the gender and racial disparities in radiology and professional organizations and societies [1, 2]. Thomas et al. noted that women were historically underrepresented as presidents and honorees across Society of Abdominal Radiology (SAR), the American College of Radiology (ACR), the American Roentgen Ray Society (ARRS), and the Radiological Society of North America (RSNA) [9]. Maddu et al. noted male predominance amongst committee members in radiology societies [10]. Niu et al. demonstrated significant percent annual increase in women faculty in academic ranks and chair positions, however, there was a decreasing proportion of women with increasing academic ranks within each year of the study period suggesting attrition or lack of promotion of women radiology faculty [11].

Similarly, race-based disparity was noted in Black and Hispanic faculty members, with a compounding effects with intersectionality of race and gender [3, 6, 11–13]. One study showed declining racial and ethnic representation in clinical academic medicine across 16 medical specialties, including radiology. In most of the specialties analyzed, Blacks and Hispanics have demonstrated a statistically significant trend of worsening underrepresentation in 2016, compared to 1990, with the exception only for Black women in obstetrics and gynecology [13]. This study also showed that women remain underrepresented in many specialties, including radiology [13].

The Society of Abdominal Radiology has made a commitment to diversity, equity and inclusion, evidenced by the creation of the Committee on Diversity, Equity and Inclusion in 2020 [14]. The mission statement of the committee support the careers all SAR members, regardless of age, gender, sexual orientation, race, physical traits, faith, religion, ethnicity, practice setting and all other identities [14]. The committee strives for inclusivity at all levels, aiming to make all SAR members feel welcome, and promoting access to opportunities for career advancement through the society. One of the first goals of the committee was to analyze the current state of our society and identify opportunities for improvement [14]. Hence, the aim of this study is to assess gender and racial diversity among the plenary session speakers at the annual meetings of the society over a 5-year period prior to the inception of the SAR Committee on Diversity, Equity and Inclusion (2016 -2020).

## Materials and methods

In this retrospective analysis, brochures of the annual meetings of the Society of Abdominal Radiology were retrospectively reviewed by 2 readers (AA, BB). The study was IRB exempt due to its retrospective nature and use of publicly available materials. Details of the plenary session, including presentation title and speaker name were recorded and analyzed by the two readers for gender and race assessment.

Gender was inferred as male, female, transgender male, transgender female or gender non-binary. This assessment was based on a combination of personal knowledge, names and physical characteristics and attire in photos curated through the internet from publicly available sources such as institutional websites and publicly available social media information [15, 16]. Two lead authors performed the analysis simultaneously. This methodology has previously been employed by author researching in this arena [8–11, 16]. Differences in the cumulative proportion of male and female speakers was analyzed using unpaired *T*-test.

Similarly, race was classified as White, Black or African American, American Indians and Alaskan Natives, Asian, Native Hawaiian and Pacific Islander and Multiracial. Similar to gender data, this data was curated by reviewing publicly available sources such as institutional websites and publicly available social media information. Differences in the cumulative proportion of race identities of the speakers was analyzed using unpaired *T*-test.

Gender representation was also compared to male and female distribution among the society members, based on self-reported member profile data available to SAR. This information was compared to the average gender representation over 5 years using a chi-square test. Race distribution information for the entire society is not available for comparison at the time of publication.

Results were considered significant when *p* ≤ 0.05. Statistical analysis was performed with Microsoft Excel (Microsoft, Redmond, WA, USA).

## Results

**Fig. 1:**
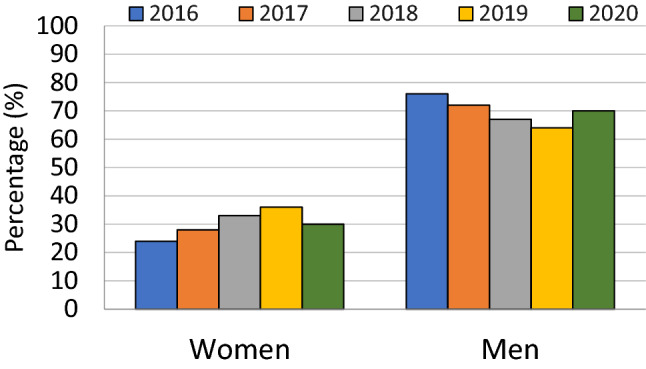
Gender representation in plenary session speakers in the Annual meetings of Society of Abdominal Radiology dating from 2016 to 2020. Proportion of women speakers has steadily increased since 2016 reaching a peak in 2019 and a slight decline in 2020

**Fig. 2:**
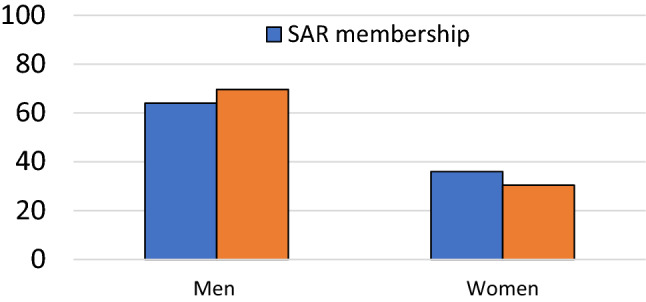
Gender representation in plenary session speakers in the Annual meetings of Society of Abdominal Radiology dating from 2016 to 2020. Average numbers of men and women speakers is compared with the membership

The gender distribution males and females over years is summarized in Table 1 and Fig. 1). Comparing the trends for gender representation of male versus female speakers, male speakers were more frequently plenary session speakers, the discrepancy being statistically significant. Over 2016–2020, on average there were [69.6% (183/263)] men plenary speakers compared to women [30.4% (80/263)] (*p*-value = 0.0007). In 2016, there were 24% women plenary speakers. This proportion was 28% in 2017, 33% in 2018 and 36% in 2019, and 30% in 2020. Historically, year 2019 saw the largest proportion of female speakers. In year 2020, the overall increased number of plenary speakers was related to a change in program format with more sessions of shorter duration compared to prior years. Although the actual number of female speakers was the highest in this year 2020, the proportion compared to male speakers declined slightly (30%) compared to 36% in 2019 (Table 1, Fig. 2). No speakers could be reliably identified as transgender, gender non-binary or gender expansive.

**Table 1:** Gender of plenary session speakers at the Annual Meeting of Society of Abdominal Radiology over 2016–2020

Gender	2016	2017	2018	2019	2020
Total speakers	45	46	46	44	82
Female	11 (24%)	13 (33%)	15 (33%)	16 (36%)	25 (30%)
Male	34 (76%)	33 (67%)	31 (67%)	28 (64%)	57 (70%)

Overall, based on self-reported data, the membership constitutes of approximately 64% male and 36% female members, with disproportionately fewer women. Compared to this, fewer women were represented in plenary speakers compared to society membership, however, this difference was not statistically significant (*p* = 0.37).

**Fig. 3:**
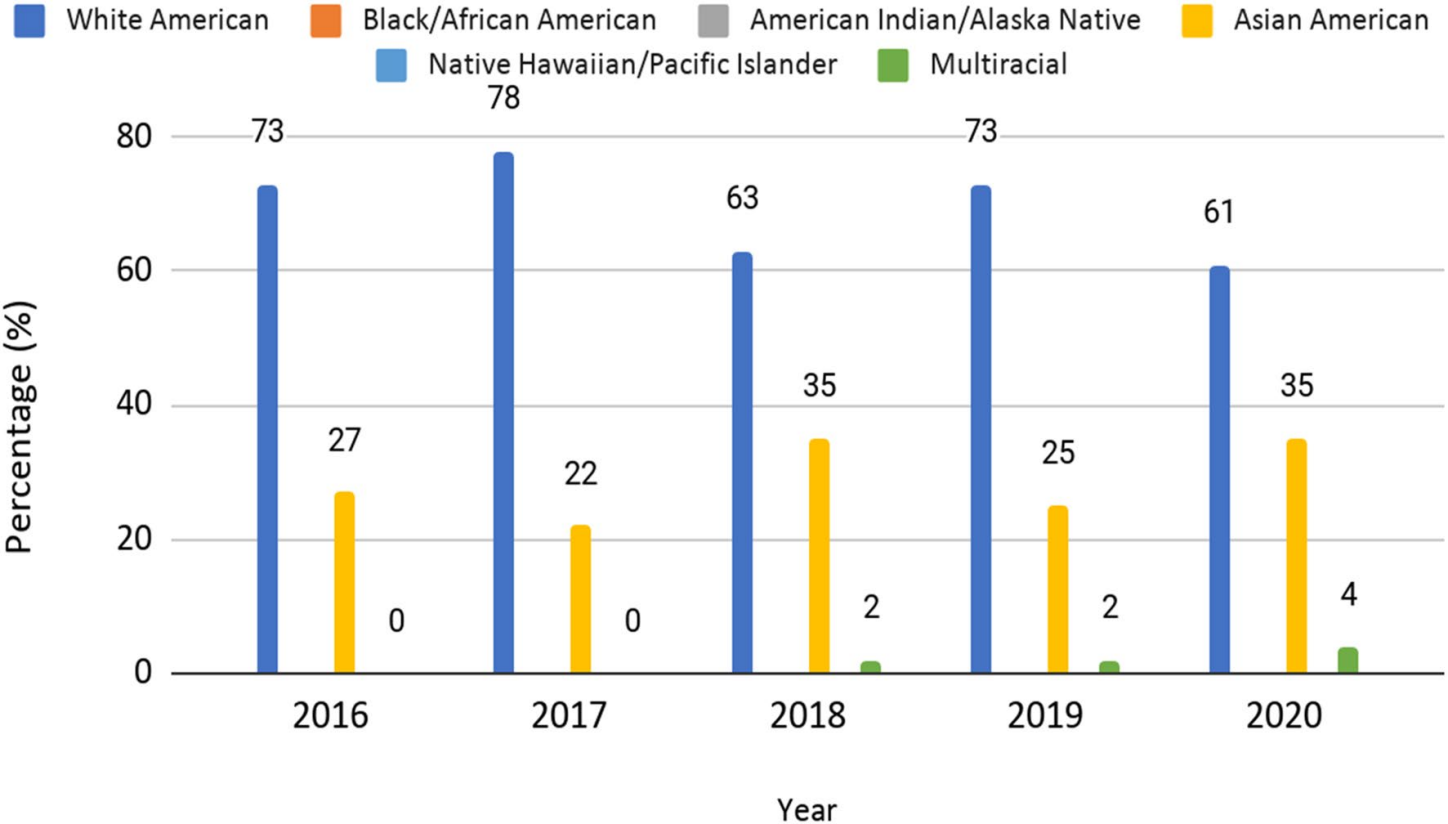
Race representation in plenary session speakers in the Annual meetings of Society of Abdominal Radiology dating from 2016–2020. White speakers were most frequently noted to be plenary session speakers, ranging above 60% of all speakers during the entire period. Representation of Asian and multiracial speakers has steadily increased over the same period. No speakers could be identified as Black or African American

The racial distribution of plenary session speakers was categorized as White, Black or African American, American Indians and Alaskan Natives, Asian, Native Hawaiian and Pacific Islander and Multiracial based on the United States census categories. The results are summarized in Table 2 and Fig. 3. When assessing racial distribution, White speakers accounted for the majority of plenary speakers, ranging from 61 to 78% (average 68.4%). Asians speakers accounted for 22 to 35% (average of 29.7%). There were no Black/African American, American Indian & Alaskan Native, Native Hawaiian & Pacific Islander plenary speakers (0%). Multiracial speakers were represented from 2018–2020, accounting for 2–4% speakers (average 1.9%) (*p*-value < 0.0001). The difference in representation of White speakers compared to other racial groups was also statistically significant (*p*-value < 0.0001). Overall, the proportion of other racial groups steadily increased reaching peak in 2020, with majority of the non-White speakers categorized as Asian.

**Table 2:** Racial distribution of plenary session speakers in the Annual meetings of Society of Abdominal Radiology dating from 2016 to 2020

	White	Black or African American (%)	American Indians (%)	Asian	Native Hawaiian (%)	Multiracial
2016	33(73%)	0	0	12 (27%)	0	0%
2017	36 (78%)	0	0	10 (22%)	0	0%
2018	29 (63%)	0	0	16 (35%)	0	1 (2%)
2019	32 (73%)	0	0	11 (25%)	0	1 (2%)
2020	50 (61%)	0	0	29 (35%)	0	3 (4%)

Self-reported race data from the society membership is unavailable at the time and hence a reliable comparison with membership racial distribution was not possible.

## Discussion

The Society of Abdominal Radiology has committed to being a diverse and equitable organization with equitable access to opportunities to all members regardless of age, gender, sexual orientation, race, physical characteristics, faith, religion, ethnicity, practice setting and all other identities. The society’s Committee on Diversity, Equity and Inclusion efforts include analysis of current trends in gender and race-based diversity of society activities and this analysis focused on gender and race representation in plenary session speakers for the annual meetings held between the years 2016 and 2020, prior to the inception of the SAR Committee on Diversity, Equity and Inclusion. Plenary sessions offer increased visibility of individuals from underrepresented groups and are an important avenue for demonstrating the society’s commitment.

Our results show the women made up a smaller proportion of plenary speakers at each of the 5 annual meetings evaluated prior to 2020. When averaged over the 5-year period, women accounted for 30.4% (80) of the 263 total speakers. Women account for 36% of the overall society membership. Thus, our results indicate that women have been underrepresented as plenary speakers, although the difference compared with overall membership did not reach statistical significance in our analysis. We were unable to reliably identify any of the presenters as transgender, gender non-binary or gender expansive. This data identifies potential area our society can focus efforts, making a conscientious effort to offer females, transgender, gender non-binary and gender expansive speakers the opportunity to present in plenary sessions. Speaker diversity promotes wider recruitment by providing audience members with successful role models in the field and enhances the careers of the speakers as well [8, 17]. Additionally, transgender and gender expansive members of the society should be encouraged to self-identify and work with the diversity committee to identify avenues for participation in the society’s activities [15].

Our results are similar to other studies that have been published analyzing representation of women in plenary session speakers for conferences and medical society meetings. Arora et al. analyzed 8535 sessions with 23,440 speakers across 98 conferences and women accounted for only 30% speakers [16]. Larson et al. evaluated speaker gender disparity in medical specialty conferences from 2013 and 2017 and found that only about 25% of speakers were women and these differences were significant when compared with physician workforce data available from Association of American Medical Colleges' (AAMC) [18]. Similar trends are observed in conferences of other specialties such as surgery, orthopedic surgery, critical care and urology [19–21]. Ghatan et al. studied the gender representation trends in Society of Interventional Radiology and found that targeted interventions such as having a woman as a session coordinator increased female speaker participation, suggesting that the inclusion of more women as coordinators is a potential mechanism for achieving gender balance at scientific meetings [8].

Based on our race analysis, White speakers were more frequently plenary speakers compared to other ethnicities over the 5 years analyzed in this study. The next most frequent race was Asian, although, there was a substantial difference in the proportion of Asian compared to White speakers. A small and increasing representation of multiracial plenary speakers was noted over 2018 to 2020. No plenary speakers could be identified as Black or African American. Self-reported race data from the society membership is unavailable at the time and hence a reliable comparison with membership racial distribution was not possible. This identifies another area where focused efforts are needed to increase involvement of URM in SAR. Pipeline issues can be particularly important here to increase the engagement of URM with SAR activities starting at early stages of their careers [17, 22, 23].

Overall, establishing a diversity and inclusion committee is a promising effort by SAR; as Prabhu et al. noted a lack of public support of membership diversity by many North American radiology societies, especially those with fewer members. As noted in this publication, the SAR Committee on Diversity, Equity and Inclusion has a publicly accessible diversity mission, with identified leaders and committee members [24]. Per Prabhu et al., identified "diversity leaders" can serve as models for societies aiming to establish their commitment to diversity and inclusion [24]. The committee notes active members on its website, including additional resources on career development, leading diverse teams, health care disparities and how to get involved with SAR.

Limitations of our study include the lack of self-reported data and demographics assessement by the authors, which is subject to bias. However, this methodology has been previously employed by other authors performing similar research [16]. No speakers could be reliably identified as transgender, gender non-binary or gender expansive. Overall, unfortunately, there is very little information available on inclusion of transgender or gender expansive individuals in radiology [15]. Similarly, race characteristics were assessed by reviewers and not self-reported by the plenary session speakers. This can certainly introduce bias in the presented data. The most accurate methodology will be to collect self-reported data from SAR members and speakers. The SAR has initiatives in place to gather this data at the time of membership renewal and speaker confirmation. However, self-reporting is not mandatory to allow people the necessary freedom to choose how to report their demographic identifiers. This indeed is a work in progress and this initial analysis will allow us to identify deficiencies of current identification processes and allow for a framework to build an equitable society in the future. The authors also acknowledge that current United States census categories may not be entirely complete and can be modified in the future as the knowledge on diversity expands.

For comparison with the overall demographics of our society, only gender-based data is currently available. Race information is unavailable. The committee is aware of this limitation and as detailed above has launched efforts to gather self-reported data on gender, race and ethnicity. This has been included in the questionnaire associated with the annual membership renewal process and at the time of speaker confirmation. Going forward plenary session speakers will be asked to provide their demographic identifiers.

## Conclusion

In conclusion, equitable gender and race-based representation of speakers in plenary sessions is important to both advance the careers of the speakers and provide role models to under-represented demographics within SAR. This also opens greater avenues for providing race and gender conscious medical education and eventually potentially translates into race and gender conscious patient care. Although current gender and race-based representation is disproportionately male and White, recent trends show increasing representation of women and URM faculty. The SAR Committee on Diversity, Equity and Inclusion has been created with the vision to identify and ameliorate differential representation in the society efforts and activities. With the data provided by this committee, our society can now work on implementing strategies to increase the representation of all genders and races in the society’s plenary sessions moving forward.
